# Somatic embryogenesis and genetic transformation in peony: challenges and applications

**DOI:** 10.3389/fpls.2026.1823513

**Published:** 2026-07-01

**Authors:** Linlan Fu, Yongliang Wang, Jiawei Jiang, Zhihong Zhang

**Affiliations:** 1 College of Public Utility, Jiangsu Urban and Rural Construction Vocational College, Changzhou, China; 2 Engineering Research Center Program of Development & Reform Commission of Jiangsu Province, Changzhou, China; 3 China Construction Bank Training Center, East China Institute, Changzhou, China

**Keywords:** efficient propagation, micropropagation, *Paeonia*, somatic embryogenesis, transgenic

## Abstract

*Paeonia*, a traditional ornamental flower and economically important crop, requires efficient propagation and targeted breeding for sustainable industrial application. Somatic embryogenesis provides a potentially transformative method for regenerating and genetically transforming *Paeonia*, offering an alternative route for rapid propagation and precision breeding. However, SE application in *Paeonia* remains challenging due to strong genotype dependence and tissue culture recalcitrance. This review summarizes recent progress in understanding the key factors affecting SE efficiency, including the decisive role of genotype, explant selection, plant growth regulator combinations, and culture conditions. Critical bottlenecks limiting large-scale application are discussed, particularly browning and hyperhydricity. Additionally, this review covers advances in genetic transformation technologies for *Paeonia*, including both stable transformation via *Agrobacterium*-mediated systems and transient transformation approaches such as *Agrobacterium* infiltration and virus-induced gene silencing (VIGS) for functional genomics studies. Although significant progress has been achieved, key challenges remain including low regeneration efficiency, incomplete transformation systems, and genotype-dependent recalcitrance across most commercial cultivars. We identify critical bottlenecks and propose that future research should integrate optimized culture systems with emerging gene editing technologies to facilitate the development of new *Paeonia* cultivars, thereby enhancing industrial scalability and market competitiveness.

## 1 Introduction

*Paeonia* species are highly valuable ornamental plants that are distributed primarily in temperate regions in Asia, southern Europe, and western North America (Beruto and Curir, 2007). The genus is classified into three subgenera with distinct biological characteristics: subgenus *Paeonia* (herbaceous peonies), including sections *Paeonia* (Eurasian herbaceous peonies, e.g., *P. lactiflora*) and *Othonnae* (Mediterranean herbaceous peonies), with nonwoody stems that die annually; subgenus *Moutan* (tree peonies), consisting of sections *Moutan* (woody tree peonies, e.g., *P. suffruticosa*, *P. rockii*, and *P. ostii*) and *Delavayanae* (subshrubby peonies), with perennial woody stems; and subgenus *Onaepia*, a small clade with only two North American species (*P. brownii* and *P. californica*) (The Plant List Database, https://powo.science.kew.org/). Beyond their ornamental value with elegant flowers and appealing colors, certain Paeonia species have significant medicinal and economic value. For example, P. lactiflora root (commonly known as “Bai Shao”) is a traditional Chinese medicine, and P. ostii seeds are rich in paeoniflorin, paeonol, and unsaturated fatty acids (UFAs), which are widely used in medicine, functional food, and cosmetics (Wang H. et al., 2023; Li P. et al., 2021; Zhang X. et al., 2019). These high-value applications have driven increasing market demand for Paeonia products globally (Kamenetsky and Yu, 2022).

However, traditional peony breeding has several limitations, including a long cycle, complex genetic background, and the resulting difficulty in the rapid accumulation of superior traits through conventional crossing. Therefore, optimizing propagation and cultivation techniques and establishing an efficient breeding system have become core areas of research. Among available techniques, somatic embryogenesis (SE) serves as a core biotechnology in plant tissue culture, providing essential support for large-scale *Paeonia* propagation and molecular breeding (Desai et al., 2022; Mohammadi et al., 2023; Williams and Maheswaran, 1986). Compared with other woody ornamental genera for which high-frequency SE and stable genetic transformation have been achieved, Rosa with SE propagation coefficient exceeding 100% in *Rosa hybrida ‘Carola’* (Duan et al., 2024), *Malus* spp. with optimized *Agrobacterium*-mediated transformation protocols (Lin et al., 2025), and the palm species *Euterpe precatoria* Mart with efficient SE systems (Ferreira et al., 2022), *Paeonia* species exhibit exceptional recalcitrance to *in vitro* manipulation. The highest reported embryogenic callus (EC) induction rate for Paeonia reaches 81% in the cultivar ‘Feng Dan’. Most commercial cultivars show EC induction below 30% (**Table 1**). This recalcitrance likely reflects three factors: high phenolic content in woody tissue, genus-specific phytohormone sensitivities, and high heterozygosity of commercial cultivars (Song et al., 2023; Beruto et al., 2004; Wang and van Staden, 2001).SE is the process by which somatic cells develop into new individuals without cell fusion, mimicking the developmental trajectory of zygotic embryos (Deo et al., 2010; Sun et al., 2014). This technique plays a pivotal role in plant regeneration and genetic transformation, facilitating the introduction of novel traits and enhancing the efficiency of tissue culture-based propagation (Zheng et al., 2024). SE is primarily classified into two types: direct SE, wherein somatic cells differentiate into embryoids without an intervening callus phase, and indirect SE, which requires an intermediate EC phase. Direct SE has been reported in certain *Paeonia* cultivars—particularly *P. rockii* and *P. ostii* ‘Feng Dan’ (**Table 1**)—but remains limited to a narrow range of genotypes. In contrast, indirect SE is documented across most Paeonia species, including P. suffruticosa, *P. rockii*, *P. ostii*, *P. lactiflora*, and Itoh peony ‘Julia Rose’ (**Table 1**), and is the primary pathway used for large-scale propagation and genetic transformation (Fan and Sun, 2024). Critical bottlenecks common to all *Paeonia* species include strong genotype dependence, explant browning, hyperhydricity (incidence up to 100% in some cultivars; Wen et al., 2019), and general tissue culture recalcitrance. These challenges collectively indicate that a comprehensive, genotype-independent regeneration system has not yet been established for the genus.

**Table 1:** Key parameters influencing somatic embryogenesis efficiency in *Paeonia* spp.

Genotypes	Explants	Basic media	Cytokinin (mg·L^-^¹)	Auxin (mg·L^-^¹)	Other additives or growth regulators (mg·L^-^¹)	Culture conditions		From	Callus induction	SE induction	Bud	Reference
*P. rockii*	Zygotic embryos	WPM	0.5 BA		0.5 GA_3_	under a dark, 4°C condition		DE	/	37.8%	5	Du et al., 2020
*P. ostii‘Feng Dan’*								DE	/	22.56%	4.67	
*P. ostii‘Feng Dan’*	leaves	WPM	1.0 CPPU	1.0 TDZ	/	under a 23°C 16 h photoperiod 25 µmol m^-2^ s^-1^ condition		DE	/	66.67%	4	Fan et al., 2024
*P. ostii‘Feng Dan’*	nodules	WPM	1.0 CPPU	0.5 TDZ	/			DE	/	41.67%		
*P. ostii* ‘Fengdan’	Meristematic nodules	mMS	1.0 CPPU	1.0 NAA	/	under a 24°C 16 h photoperiod 50 µmol m^-2^ s^-1^ condition		IE	81.25%	68.75%	13.40	Xu et al., 2022
*P. ostii* ‘Fengdan’	Mature embryo	WPM	1.0 BA	/	0.5 GA3, 3.0 phytagel	under a 25°C 12 h photoperiod 25-30 mmol m^-2^ s^-1^ condition		IE	100%	/	5.4	Liu et al., 2022
*P. ostii* ‘Fengdan’	Zygotic embryo, cotyledon	MS	3.0 BA	1.0 NAA	/	under a dark, poikilothermic (24 °C for 16 h and 18 °C for 8 h)		IE	86.0%, 56%	about 70%, 60%	/	Ren X. et al., 2019
	Hypocotyl	WPM						IE	82%	about 50%	/	
*P. ostii* ‘Fengdan’	Cotyledon	MBP	0.25 TDZ	0.5 2.4-D	1.0 AC	under a dark, 23°C condition		IE	31.61%	/	/	Zhang et al., 2023a
*P. ostii* ‘Fengdan’	Zygotic embryos	MS	3.0 BA	1.0 NAA	/	under a dark, poikilothermic (24 °C for 16 h and 18 °C for 8 h) condition		IE	57.88%	87.5%	8.8	Ci et al., 2022
*P. lactiflora* ‘Fengdanbai’	Young flower petals	MS	3.0 BA	2.0 2,4-D+ 0.3 NAA	/	under a 24°C 14 h photoperiod 30-50 mmol m^-2^ s^-1^ condition		IE	98.52%	34.81%	23.4	Chen et al., 2022
*P. lactiflora* ‘Fengdanbai’	Cotyledon	MS	0.3 TDZ	3.0 2,4-D+0.1 NAA	3.0 AC	under a dark, 23°C condition		IE	100%	94.17%	4.77	Zhu et al., 2022
*P. lactiflora* ‘Fenyu nu’	Zygotic embryos	1/2 MS	1.0 BA	/	1.0 GA3	under a 25°C 16 h photoperiod 50 mmol m^-2^ s^-1^ condition		IE	95.33%	89.43%	5.56	Shen et al., 2014
*P. suffruticosa* Andr.	Leaves /petioles	MS	/	1.0 2,4-D	/	under 25°C 16 h photoperiod	100% blue CCFL or 100% red CCFL, 45 mmol m^-2^ s^-1^	IE	90.0% /73.3%	/	/	Wang H. et al., 2010
							dark	IE	66.7% /90.0%	/	/	
*P. suffruticosa* cv. Kao	Petiole	1/2 MS	/	2.0 2,4-D	2 AgNO_3_, 1.0 PVP	under a dark, 25°C condition		IE	88%	/	/	Gao et al., 2020
Itoh peony cv.‘Julia Rose’	Axillary buds	1/2 MS	2 .0 BA	/	0.5 ferulic acid, 0.1 AC	under a 25°C 16 h photoperiod 45 mmol m^-2^ s^-1^ condition		IE	unquantified	unquantified	unquantified	Boltenkov et al., 2016

2,4-D - 2,4-dichlorophenoxyacetic acid; BA - N6-benzyl adenine; CCFL - cold cathode fluorescent light; CH - casein hydrolysate; GA3 - gibberellic acid; IAA - indole-3-acetic acid; IBA - indole-3-butyric acid; KT - kinetin; LH - lactoalbumin hydrolysate; MS medium - Murashige and Skoog (1962); NAA - 1-naphthalene acetic acid; PVP - polyvinylenolpyruvate; TDZ - thidiazuron; WPM medium - Woody Plant Medium; Vc - Vitamin C; AC activated carbon; MBP medium - sucrose was replaced with glucose added in MS medium; CPPU - N-(2-chloro-4-pyridyl)-N-phenylurea; mMS medium - half-strength macroelements and full-strength Ca2^+^ MS medium; DE- Direct Embryogenesis; IE- Indirect Embryogenesis.

Genetic transformation, as a key molecular strategy for the targeted improvement of plant traits, has demonstrated considerable application potential in peony breeding. An efficient, stable, and reproducible receptor explant is a fundamental prerequisite for successful genetic transformation. SE, characterized by remarkable regenerative efficiency and stable genetic background, provides consistent materials for genetic transformation systems (Fan et al., 2024; Wang et al., 2020), serving as a key bridge connecting molecular technologies and *Paeonia* breeding. This technology offers innovative approaches for increasing disease resistance, precisely regulating flower color, and alleviating abnormal tissue culture phenomena (Choudhury and Rajam, 2021; Ramkumar et al., 2020; Zhao Q. et al., 2025). To date, stable genetic transformation in *Paeonia* has relied primarily on Agrobacterium-mediated transformation using somatic embryos or embryogenic callus as explants, whereas particle bombardment has been used primarily for transient expression assays (Fan et al., 2020; Koichi et al., 2002; Taylor and Fauquet, 2002). However, practical applications remain constrained by immature tissue culture systems and strong genotype dependence (Wang et al., 2018; Wen et al., 2016b; Wen et al., 2021).

This review systematically summarizes research progress on SE and SE-based genetic transformation in Paeonia over the past three decades. Drawing on 110 core studies, we analyze the key factors affecting SE efficiency and the major constraints hindering large-scale application. We also evaluate progress in both Agrobacterium-mediated stable transformation and transient transformation systems. Based on the review, we identify three priority areas for future research: (i) mechanistic studies to elucidate the molecular basis of recalcitrance, (ii) systematic cross-cultivar comparisons to differentiate genotype-specific barriers from general ones, and (iii) integration of gene editing with optimized SE protocols.

## 2 Factors affecting somatic embryogenesis in *Paeonia*

### 2.1 Genotype

The impact of genotype on SE efficiency has been widely reported in various plant species (Naranjo et al., 2015; Molina et al., 2002). In contrast, other woody ornamental species, such as rose and apple, typically yield reproducible protocols across cultivars from responsive genotypes, whereas Paeonia exhibits extreme cultivar specificity, often failing to transfer SE protocols between cultivars (Karami and Saidi, 2010). Some cultivars of tree peony, such as *P. rockii* and *P. ostii* ‘Feng Dan,’ can be regenerated via direct SE (**Table 1**). In contrast, indirect SE, which requires an intermediate embryogenic callus (EC) phase, is more common in most *Paeonia* species, including tree peony cultivars such as *P. rockii* and *P. ostii* ‘Feng Dan’ and *P. suffruticosa* ‘Fengdanbai’ as well as the herbaceous peony *P. lactiflora* and Itoh peony cv. ‘Julia Rose’ (**Table 1**), which often follow this pathway under conventional conditions, in which their somatic cells first dedifferentiate into a callus before differentiating into embryoids.

Most *Paeonia* cultivars are natural hybrids or polyploids with high genetic heterozygosity, leading to divergent responses to hormone combinations, explant types, and culture environments. Since the emergence of SE technology, numerous studies have optimized protocols for specific cultivars—predominantly ‘Feng Dan’, ‘Feng Dan Bai’, *P. suffruticosa*, and *P. rockii* (**Table 1**). The highest EC induction rates (up to 81.25%) have been achieved in *P. ostii* ‘Feng Dan’, which exhibits relatively low genetic heterozygosity (Xu et al., 2022). However, applying the same protocol to other cultivars yields EC induction below 30%, abnormal embryo ratios exceeding 40%, and sometimes complete induction failure (Du et al., 2020). This pattern indicates that ‘Feng Dan’ represents a genotype-specific outlier rather than a model for general optimization. The optimized conditions for ‘Feng Dan’ (MS medium with 1.0 mg·L^-^¹ CPPU and 1.0 mg·L^-^¹ NAA; Xu et al., 2022) are suboptimal—or even inhibitory—for other varieties (Cai et al., 2020), reflecting narrow tolerance ranges for hormone concentrations and explant treatments. While all related studies have consistently confirmed the decisive role of genotype in the SE of *Paeonia* (Naranjo et al., 2015; Lowe et al., 2018; Fan et al., 2024; Cai et al., 2020), these studies remain limited to phenotypic observations and fail to elucidate the underlying molecular mechanisms. Specifically, existing approaches are confined to local adjustments of culture media for individual cultivars. Although most studies have successfully obtained embryogenic cultures (Wang J. et al., 2010), a stable and efficient SE system for the entire genus remains elusive. Key challenges include abnormal embryo proportions exceeding 40% and complete induction failure in some cultivars. Furthermore, current studies have focused on a narrow range of easily manipulable cultivars such as ‘Feng Dan’ and ‘Feng Dan Bai’, leaving numerous commercially important cultivars, including *P. suffruticosa* ‘Luoyang Hong’ and *P. rockii* ‘Jv He San Bian’, without systematic investigation. Consequently, studies remain trapped in a repetitive cycle of cultivar-specific optimization, failure to generalize, and reoptimization for individual cultivars, which has yet to enable a substantive breakthrough for the large-scale application of SE technology in *Paeonia*.

### 2.2 Explant selection

The successful application of SE relies on the induction stage, during which the effective induction of somatic embryos is significantly influenced by explant selection (Zhang et al., 2023a). In *Paeonia* plants, various parts, such as seeds, axillary buds, leaves, petioles, and embryos, can be used as explants to induce somatic embryos (da Silva et al., 2012), but the efficiency varies significantly among different explant sources. Among tree peonies, underground buds have demonstrated superior performance, achieving SE induction rates of 65–80% in ‘Feng Dan’ (Meng, 2011), whereas most herbaceous peony protocols rely on seeds as explants. For *P. lactiflora* ‘Feng Dan Bai’, seeds collected 90 days after flowering exhibited the highest germination rate (94.17%), while seeds collected 70 and 80 days after flowering also yielded high embryo induction rates (100%) (Zhu et al., 2022).

This variation in efficiency reflects the fact that explant source is a primary determinant of SE success in *Paeonia*, with immature or embryonic tissues consistently outperforming mature differentiated organs (Malabadi et al., 2025). Studies have shown that cotyledons from aseptic seedlings exhibit the greatest ability to induce and proliferate because of their active growth stage and high activity of meristematic cells (**Table 1**). In contrast, highly differentiated organs from adult plants (e.g., mature stems and fully expanded leaves) exhibit significantly lower EC induction efficiency because of their reduced developmental plasticity (Woo et al., 2021; Chen et al., 2022, 2001). This pattern has been verified in different *Paeonia* species. In *P. lactiflora*, indirect SE was carried out using cotyledons, hypocotyls, and embryos isolated from sterilized seeds as explants. The callus induction rate of cotyledon explants reached as high as 98.89%, which was superior to that of hypocotyl and embryo explants (Ren X. et al., 2019). Zhu et al. (2022) also confirmed that cotyledons were the most suitable explants for SE in *P. suffruticosa*. Thus, cotyledons not only have a low contamination rate but also exhibit strong differentiation and proliferation abilities, making them the optimal choices for EC induction and proliferation.

The superior performance of cotyledons can be attributed to their early developmental stage and undifferentiated meristematic cells, which possess strong division capacity and a low dedifferentiation threshold (Malabadi et al., 2025; Liu et al., 2022; Ren et al., 2020). Additionally, cotyledons exhibit low contamination rates and strong differentiation and proliferation abilities, making them optimal for EC induction and proliferation. Further optimization of the direct SE pathway in ‘Feng Dan’ demonstrated that quarter-embryo explants yielded significantly higher EC induction rates and bud proliferation coefficients (5.4 ± 0.2) compared to intact or half-embryo configurations (Liu et al., 2022), suggesting that spatial confinement of embryogenic cells enhances inductive efficiency.

### 2.3 Plant growth regulators

The most commonly used induction protocol for SE in *Paeonia* plants involves the induction of callus formation in medium supplemented with auxins, followed by the transfer of the callus to medium containing low concentrations of auxins or one in which the hormone ratio is adjusted to increase the proportion of cytokinins to promote somatic embryo development (**Table 1**). That is, the establishment and maintenance of somatic embryo culture systems for almost all plant species are centered on the precise regulation of PGRs. Common somatic embryo-inducing PGRs include auxins, such as 2,4-D, indole-3-acetic acid (IAA), and NAA, as well as cytokinins, such as 6-benzylaminopurine (6-BA), TDZ, and kinetin (KT) (Malabadi et al., 2025). In the herbaceous peony hybrid ‘Fen Yunu’ ♀ × ‘Fen Yulou’ ♂, clear differences were observed among the different hormone treatments. When treated with 1.0 mg·L^−1^ NAA, the calli induced from cotyledon and hypocotyl explants were dense and pale yellow and exhibited vigorous growth, whereas those derived from embryonic bases were in an expanded state and consisted of two types, namely, dense pale yellow and transparent watery structures. When treated with 2.0 mg·L^−1^ 2,4-dichlorophenoxyacetic acid (2,4-D), the calli from cotyledons and hypocotyls exhibited a white flocculent surface with a soft and fragile texture, whereas those induced from embryo explants maintained excellent morphology, being dense, pale yellow and transparent. Compared with NAA and 2,4-D, treatment with 2.0 mg·L^−1^ picloram (PIC) had significantly worse effects; not only was the callus induction rate of the embryo explants relatively low, but the occurrence position of the calli was also irregular (Song et al., 2023). These findings demonstrate that hormone type and concentration play critical roles in determining both SE efficiency and tissue morphological characteristics in *Paeonia*.

The regulatory differences among the aforementioned single hormones suggest that the improvement in SE induction efficiency requires further optimization of hormone combinations. This idea has been confirmed by studies on different species and cultivars of *Paeonia*. The induction rate of an EC is relatively low when auxin-type PGRs are used alone, while a combination of auxins and cytokinins can significantly increase induction efficiency (Malabadi et al., 2025). Moreover, the optimal PGR combinations vary among tree peony and herbaceous peony cultivars. Across all reported PGR combinations, a consistent pattern emerges: auxin–cytokinin combinations are superior to single-hormone treatments, with optimal ratios ranging from 1:1.4 to 1:5 (auxin:cytokinin) for EC induction (Cheng et al., 2013). However, the specific PGR identities that maximize efficiency vary substantially among cultivars—for example, CPPU (a synthetic cytokinin) outperforms 6-BA in ‘Feng Dan’ but not in other varieties (**Table 1**). This cultivar specificity suggests that hormone response pathways are modulated by genotype-specific factors, likely including the differential expression of hormone receptors and downstream signaling components. In summary, the rational application of PGRs and the precise regulation of the ratio of auxins to cytokinins are not only decisive factors for the successful induction of somatic embryos in *Paeonia* but also core technical links to improve the occurrence rate of somatic embryos in tissue culture.

### 2.4 Culture conditions

**Figure 1:**
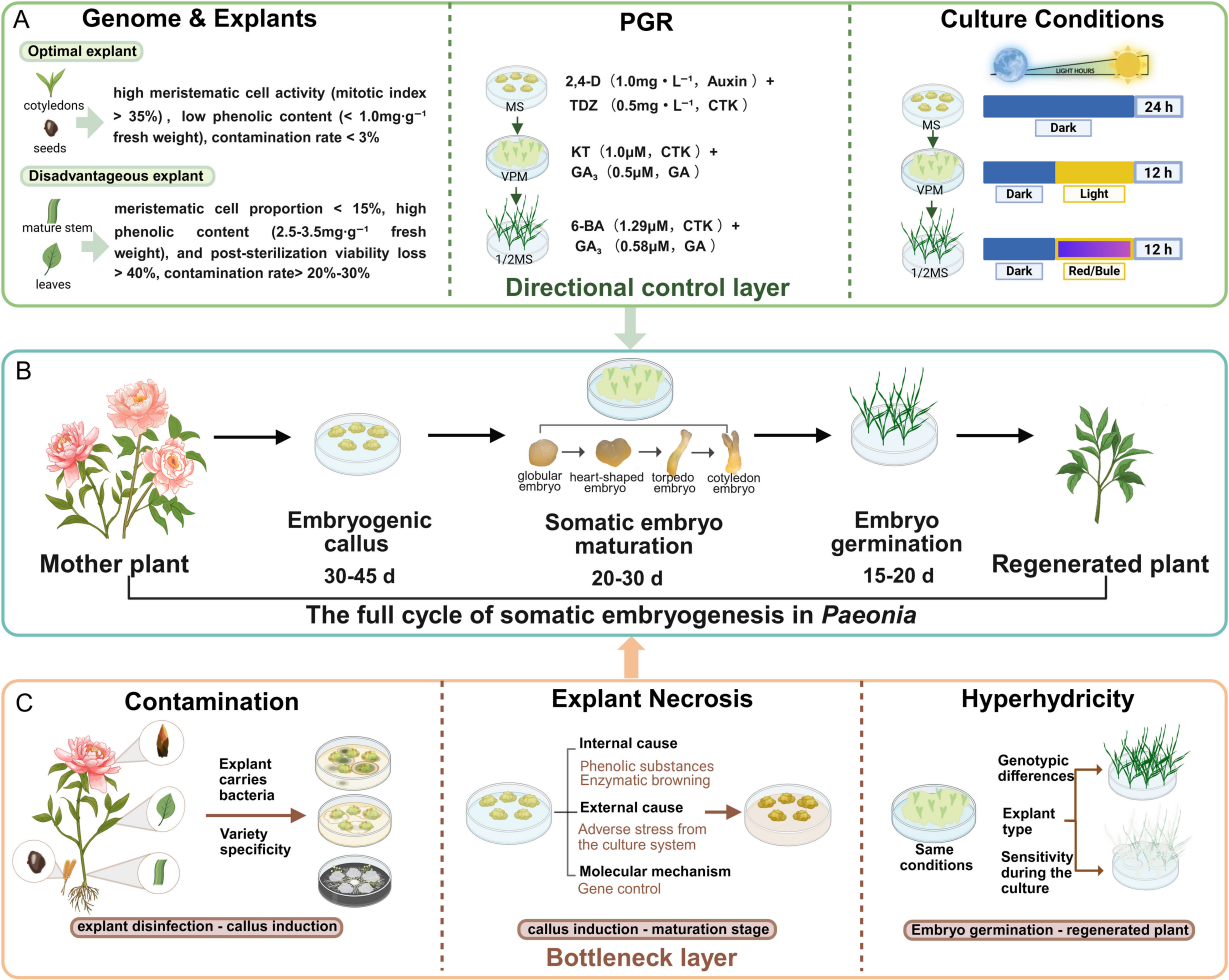
Directional control layer, full-cycle process and limiting factors for SE in *Paeonia.* **(A)** Directional control layer of somatic embryogenesis (SE) in *Paeonia*; **(B)** full-cycle process of SE in *Paeonia*; and **(C)** bottleneck layer of SE in *Paeonia.*.

Temperature and light are key environmental regulators of plant SE. The relevant parameters, including temperature level and stability, light intensity, photoperiod, and light quality, strongly influence all stages (from EC induction and somatic embryo maturation to germination) by regulating cellular metabolic rhythms, the expression of key embryo development-related genes, and physiological and biochemical processes (Malabadi et al., 2025). These parameters must be closely coordinated with explant types and PGR combinations to maximize SE efficiency and regenerated plant quality (**Table 1**; **Figure 1A**).

From the perspective of temperature conditions, the requirements for SE exhibit distinct “stage-dependent temperature requirements”, defined as the stage-dependent uniqueness of temperature demands that are tightly coupled to the physiological and biochemical characteristics of each developmental phase, including EC induction, somatic embryo maturation, and germination. SE typically benefits from low-temperature or stable medium-temperature environments and is highly sensitive to high temperatures exceeding 28 °C, particularly in woody plants (Trontin et al., 2025; Kvaalen and Johnsen, 2007). A temperature range of 23–25 °C not only ensures the fundamental metabolic activity required for the differentiation of *Paeonia* explants such as mature embryos of *P. ostii* ‘Feng Dan’ (Boltenkov et al., 2016) and cotyledons of *P. lactiflora* ‘Feng Dan Bai’ but also promotes cell division to facilitate callus formation (Zhu et al., 2022). Moreover, this temperature can effectively inhibit the oxidation of phenolic substances that are prone to occur in *Paeonia* explants and reduce the risk of browning (Wen et al., 2025). For mature embryos of *P. ostii* ‘Feng Dan’, 23–24 °C achieves EC induction rates exceeding 80% with only 12% browning, whereas 28 °C increases browning to 35% and compromises callus texture and embryogenicity. During somatic embryo maturation, constant temperature of 24–26 °C is required; at 25 °C, the heart-shaped to cotyledon-shaped transition reaches 70% with only 8% malformation, whereas temperature fluctuations of ±3 °C increase malformation to 22%, primarily manifesting as asymmetric cotyledons and shortened hypocotyls (Zhang et al., 2023a). Importantly, the optimal temperature range coincides with the thermal minimum for phenolic oxidation, suggesting a correlative rather than causative relationship between temperature and browning prevention.

Light conditions regulate SE through the synergy of three key parameters, namely, light intensity, photoperiod and light quality, and the requirements for these parameters vary significantly across different developmental stages. The EC induction stage typically requires low-light environments at 25–50 μmol·m^-^²·s^-^¹ (Ci et al., 2022; Liu et al., 2022; Gao et al., 2020). For some *Paeonia* species, initial dark culture followed by low-light transfer is optimal. For example, *P. lactiflora* ‘Feng Dan Bai’ benefits from 15 days of initial dark culture followed by low light (Zhu et al., 2022), which meets light signal requirements for dedifferentiation while preventing callus elongation and fibrosis from prolonged darkness. Total dark culture during SE induction also enhances EC formation: mature embryos of *P. ostii* ‘Feng Dan’ cultured in total darkness at 24 °C achieve induction rates exceeding 85%, significantly higher than under light culture (Ci et al., 2022). For species sensitive to light quality, alternating red–blue light is preferable. In *P. suffruticosa*, 80% red and 20% blue light at 45 μmol·m^-^²·s^-^¹ significantly improves SE induction (Zhao, 2021). Similarly, in *P. rockii* ‘Jing Hong’, 70% red and 30% blue light at 25 °C increases EC induction by 28% compared to white light, with embryogenic cell proportions exceeding 75% (Du et al., 2020).

In summary, the efficiency of SE in *Paeonia* is governed by four key factors. Genotype is the most critical determinant, with cultivars such as ‘Feng Dan’ achieving EC induction rates up to 81%, while most commercial cultivars such as *P. suffruticosa* and *P. lactiflora* remain below 30% or have no established SE protocols, and other *P. rockii* cultivars such as ‘Jv He San Bian’ show similarly low efficiency. Explant selection is equally important, as cotyledons from immature seeds consistently demonstrate the highest induction efficiencies (up to 98.89%) due to their high meristematic activity. PGRs, particularly auxin-cytokinin combinations at ratios of 1:1.4 to 1:5, are essential for callus induction and somatic embryo development, though optimal PGR combinations vary among cultivars. Culture conditions, including temperature (23–25 °C), light (low intensity or dark culture initially, with red-blue light supplementation), and basal media (MS or WPM), further modulate SE efficiency. Together, these factors interact to determine SE outcomes in Paeonia. However, this strong genotype dependence suggests that a genotype-independent regeneration system has yet to be established for the genus.

## 3 Key constraints in somatic embryogenesis in *Paeonia*

### 3.1 Sterilization and browning

The SE of Paeonia typically begins with obtaining explants from greenhouse-cultivated or outdoor plants, or directly from seeds. These materials often carry surface microorganisms, making disinfection necessary. Sampling time significantly affects disinfection efficacy. For dormant buds, the optimal sampling time is during germination in February to March of early spring, when bud dormancy has been broken and cell metabolism is active, facilitating subsequent disinfection. Delaying sampling until buds have germinated and leaves have unfolded increases surface contamination, complicating disinfection procedures (Zhang et al., 2021). Given these challenges, chemical disinfection protocols are essential for effective sterilization. Sodium hypochlorite (NaClO) is frequently used in disinfection schemes because of its wide application as a commercial bleaching agent and disinfectant, as well as its cost effectiveness. However, when NaClO is used alone, its ability to eliminate persistent microorganisms is limited, and completely removing a complex microbial community from the surface of the initial material is still challenging. Therefore, the combined use of NaClO and ethanol has become a key approach for improving disinfection efficiency. Specific parameters vary with *Paeonia* variety and explant type, typically involving 70–95% ethanol (30 sec to 2 min) followed by 0.2–2% NaClO (5–20 min) (Xu et al., 2022; Gao et al., 2020; Chen et al., 2022; Boltenkov et al., 2016). Mercury chloride, sometimes combined with ethanol for herbaceous peonies with high contamination risks (e.g., seeds and underground buds), has been largely phased out due to operator health risks, toxic residues in culture medium, and environmental pollution concerns. Through these combined disinfection techniques, sterile culture has been successfully established for various tree peony varieties.

While effective disinfection lays the foundation for Paeonia micropropagation, browning persistently limits system stability and efficiency (**Figure 1C**). Browning results from oxidation of phenolic compounds—abundantly produced as secondary metabolites in this genus—catalyzed by polyphenol oxidase (PPO) released upon cell damage. These phenolics not only are waste products but also actively participate in defense responses and are associated with differentiated cell states; their accumulation during culture may reflect a physiological conflict between dedifferentiation and defense programming (An and Zhao, 2005). This mechanistic insight suggests that browning cannot be fully resolved by antioxidant supplementation alone; Most *Paeonia* cultivars synthesize large amounts of phenolic substances during micropropagation, and tissue culture browning is positively correlated with *in vivo* PPO activity. The release of phenolic substances causes explant necrosis, directly reducing regeneration system stability (Wen et al., 2025). Several strategies have been developed to mitigate browning. Low-temperature explant collection (0–5 °C in winter or early spring) reduces initial phenolic synthesis rates (Chen, 2005), while dark culture (7–10 days during root induction) inhibits light-induced phenol oxidase (Zhu et al., 2022). Medium optimization using 1/2 modified MS or low-salt WPM with appropriate PGRs alters physiological and metabolic statuses, reducing phenolic accumulation (Du et al., 2020; Shen et al., 2014). Chemical interventions include antioxidants (ascorbic acid, melatonin, citric acid) that scavenge reactive oxygen species, and adsorbents (activated carbon, polyvinylpyrrolidone) that reduce free phenolic concentrations through physical adsorption (Gao et al., 2020). However, these methods only partially reduce browning in some cultivars or at specific stages and do not represent complete solutions for most cultivars. Future studies should employ comparative metabolomics and transcriptomics to identify cultivar-specific differences in phenolic metabolism and enable targeted interventions.

### 3.2 Hyperhydricity

In *in vitro* cultures, hyperhydricity is a typical morphological abnormality in plants and has emerged as a key factor that limits the efficiency of SE and related large-scale reproductive applications. In essence, during SE, embryogenic cells or tissues cultured *in vitro* may experience physiological metabolic disorders and morphological structural abnormalities because of excessive water accumulation (Kevers et al., 2004). Specifically, in peony SE, vitrified individuals exhibit clear and specific abnormal characteristics: the EC tissue appears loose and watery, the overall morphology of somatic embryos is abnormal, the development of cotyledons is asymmetric or semitransparent, the embryonic axis is shortened, thin-walled tissue proliferates excessively, and the number of cell layers in the leaf palisade tissue is reduced, resulting in a loose leaf mesophyll structure, which affects the distribution of photosynthetic pigments and causes leaf curling (Zhao D. et al., 2025). These structural abnormalities directly interfere with the key stages of SE and can cause the death of entire tissue-cultured seedlings, posing a direct threat to the stability of *Paeonia* tissue culture systems (Hassannejad et al., 2012; Huang et al., 2010; Sreedhar et al., 2009).

Among different tree peony cultivars, the damage caused by hyperhydricity during SE significantly differs across cultivars. The hyperhydricity rate varies drastically during the induction stage of SE, fluctuating between 0% and 76.2% (Li and Kong, 2010; Chu and Li, 1992). This difference is reflected not only in the occurrence probability but also in the fact that the hyperhydricity phenomenon in some cultivars can even completely block the process of SE. In extreme cases (e.g., all 8 tested *P. rockii* cultivars; Wen et al., 2019), hyperhydricity completely prevents SE progression. Even under mild hyperhydricity, the consequences are devastating: in *P. ostii* ‘Feng Dan’, somatic embryo maturation decreases to 42%, germination to 11%, and plant regeneration to 0% (Ren X. et al., 2019). This bottleneck underscores the urgent need to understand the physiological and molecular basis of hyperhydricity—which remains poorly characterized—to develop genotype-independent solutions.

Three strategies have been explored to reduce hyperhydricity incidence: environmental regulation, medium optimization, and hormone regulation. For environmental regulation, stable culture temperature at approximately 25 °C and light intensity of 50–100 μmol·m^-^²·s^-^¹ provide a stable environment for somatic embryo maturation (Wen et al., 2019; Li and Kong, 2010; An and Zhao, 2005). For medium optimization, MS medium with a 3/4 reduction in ammonium nitrate content effectively alleviates metabolic disorders induced by excessive nitrogen, reducing hyperhydricity by 28% (Cai et al., 2020). Increasing agar concentration by 30% to regulate osmotic pressure reduces hyperhydricity to less than 15%, enabling successful somatic embryo seedling induction (Wen et al., 2019). For hormone regulation, reducing 6-BA concentration in *P. ostii* ‘Feng Dan’ culture prevents looseness of embryonic tissues caused by excessive cell division, achieving SE efficiency of 58% (Zhang K. et al., 2019). Despite these efforts, SE and plant regeneration efficiencies remain substantially below ideal levels, and none of these methods can fundamentally resolve hyperhydricity restriction (Wen et al., 2019). The persistent failure to resolve hyperhydricity in Paeonia SE reflects a deeper problem: current understanding remains descriptive rather than mechanistic. We know that environmental and hormonal factors modulate hyperhydricity incidence, but we do not understand why certain cultivars are inherently susceptible or how excessive water accumulation disrupts embryogenic development at the cellular level.

### 3.3 Maturation and regeneration

During the process from somatic embryo maturation to complete plant regeneration in *Paeonia*, shoot proliferation and root induction are the core links of the regeneration system. This process remains the lowest-efficiency step for many *Paeonia* cultivars, among which the type and concentration of PGRs are the main factors that affect SE (Ali et al., 2022; Wang J. F. et al., 2010; Niu et al., 2025). Studies on the direct embryogenesis pathway have focused mostly on woody peonies, and there are no reports in the literature of achieving plant regeneration in herbaceous peonies through this pathway. The type and concentration of cytokinins can regulate the direct embryogenesis pathway, and high levels of cytokinins are key factors that maintain the efficient progression of the differentiation process (Duan et al., 2022). Xu et al. (2022) demonstrated in their study on *P. ostii* ‘Feng Dan’ that the combination of 0.29 mg·L^-^¹ 6-BA and 0.20 mg·L^-^¹ GA_3_ yielded the optimal induction effect, with the average number of adventitious buds reaching 16.03, the rooting rate reaching 43.33%, and the survival rate reaching 45.83%. In WPM medium supplemented with 0.5 mg·L^-^¹ CPPU and 0.5 mg·L^-^¹ TDZ, approximately 4 regenerated buds per explant, 50% rooting rate, and 40% survival rate were achieved. Notably, BA alone failed to induce adventitious buds (Fan et al., 2024). Additionally, 0.5 mg·L^-^¹ BA combined with 0.5 mg·L^-^¹ GA_3_ increased germination rate to 45% (Du et al., 2020). This dissociation between shoot and root development suggests that hormonal conditions optimal for morphogenesis are not conducive to rhizogenesis—a problem potentially reflecting inadequate endogenous reserves in somatic embryos. The cultivar-specific optimal combinations (proliferation coefficients ranging from 2.20 to 10.98; **Table 1**) indicate that downstream signaling components, not merely hormone availability, limit morphogenic outcomes.

In addition to medium components, structural characteristics during SE also significantly affect bud differentiation efficiency, with substantial differences in surface structure between calli with low and high bud differentiation rates. During the SE of the herbaceous peony ‘Feng Dan Bai’, Chen et al. (2022) reported that there were two types of surface structures in calli: those with high differentiation rates exhibited completely smooth protrusions arranged in a dense pattern, whereas those with low differentiation rates had smooth protrusions (usually with cracks at their bases) and cracks. These structures may greatly restrict the development of bud primordia, making it impossible to achieve a high bud differentiation rate even under optimal culture conditions. Therefore, when calli are selected for further differentiation and culture, in addition to removing browned parts and selecting healthy specimens, calli with different structures should be processed separately. Choosing calli with distinct and densely arranged protrusions for further culture can significantly improve bud differentiation efficiency (Zhao D. et al., 2025). In *P. rockii* ‘Jv He San Bian’ and ‘Jing Hong’, although the differentiation rate of embryonic calli increased after they were transferred to differentiation medium, some of them still failed to differentiate normally (Du et al., 2020). During development, these calli undergo malformation and browning, resulting in the inability to differentiate further. Moreover, there was a widespread imbalance between root and bud development among the calli that can differentiate into buds; some calli could only differentiate into buds and could not be induced to form roots after being transferred to rooting medium, ultimately failing to develop into complete plants (Zhu et al., 2011). This phenomenon may be attributed to internal physiological imbalance caused by excessive exogenous growth regulators, insufficient key metabolite accumulation, and premature bud differentiation.

## 4 Genetic transformation via somatic embryogenesis in *Paeonia*

### 4.1 Stable genetic transformation

**Figure 2:**
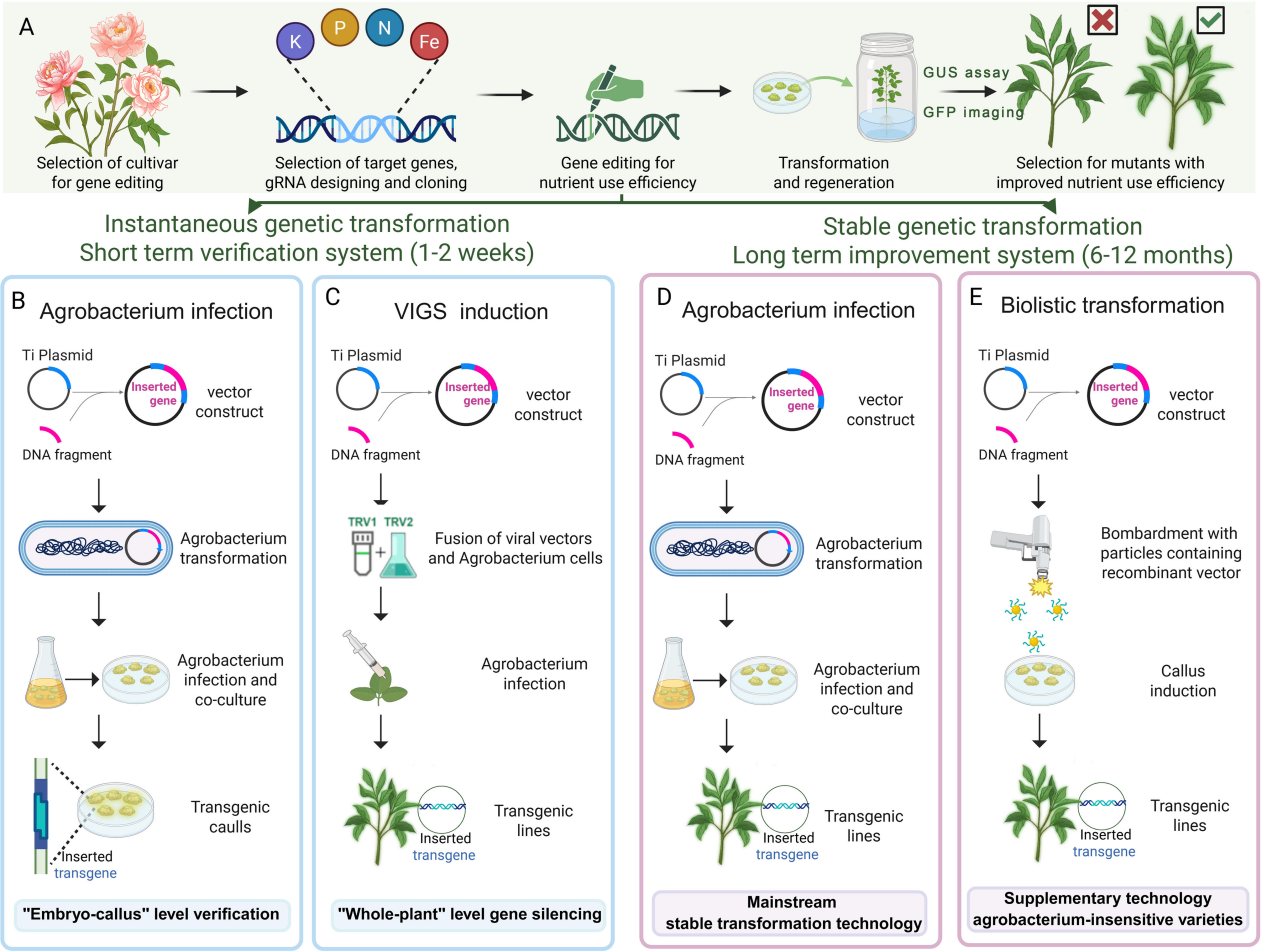
Genetic transformation systems in *Paeonia.* **(A)** Genetic transformation in *Paeonia*; **(B)** *Agrobacterium*-mediated transient genetic transformation method; **(C)** virus-induced transient gene silencing; **(D)** *Agrobacterium*-mediated stable genetic transformation method; and **(E)** particle bombardment method.

The ornamental and medicinal value of *Paeonia* species have attracted researchers to conduct in-depth and systematic investigations on these plants. With the completion of the chromosome-level genome assembly of *Paeonia* plants (Liu et al., 2019), the scope and depth of research on molecular mechanisms related to their genetic improvement have greatly expanded (Luan et al., 2024; Yuan et al., 2022; Li L. et al., 2021; Dayal et al., 2003). However, issues such as low transformation efficiency and difficulties in establishing regeneration systems and in stably integrating exogenous genes into the genome of *Paeonia* plants remain core technical bottlenecks that urgently need to be addressed (Huang et al., 2009; Wang H. et al., 2025). Stable and transient genetic transformation methods, including *Agrobacterium*-mediated transformation, virus-induced transformation, gene guns, and other transformation approaches, are commonly used in various plants (**Figure 2**; **Table 2**). To date, two major technical approaches—*Agrobacterium*-mediated transformation and particle bombardment—have been developed for the stable genetic transformation of *Paeonia* using somatic embryos (**Figures 2D, E**). Owing to differences in their mechanisms of action, these two methods significantly differ in terms of transformation efficiency, applicability, and genetic stability. *Agrobacterium*-mediated transformation is the preferred method for stable gene integration in *Paeonia* because of advantages such as low copy number insertion (typically 1–2 copies) and high genetic stability (Hensel, 2020; Xu et al., 2017; Singh et al., 2016). However, these advantages are theoretical rather than practical: actual transformation efficiency remains extremely low, with only *P. ostii* and *Paeonia × lemoinei* ‘High Noon’ exhibiting stable transformation events and conversion rates as low as 1.33% (Wei, 2018). This gap between theoretical promise and empirical failure highlights the need to understand host immune responses and epigenetic barriers to T-DNA integration in *Paeonia*. Particle bombardment offers an *Agrobacterium*-independent route for gene introduction and has been used primarily for transient expression assays prior to stable transformation attempts (Fan et al., 2020). However, for stable integration, this method yields efficiencies less than 2% with frequent multicopy insertions (3–5 copies), increasing the risks of gene silencing and position effects. Given these limitations, particle bombardment should be considered a preverification tool rather than a primary transformation method for *Paeonia*.

**Table 2:** Genetic transformation in *Paeonia* spp.

Plant species	Explants	Genetic transformation	Transformation method	Strains	Vector	Gene transferred	Treatment	Genetically modified traits	References
*P. ostii* ‘Feng Dan’	cotyledon	Stable Genetic Transformation	infection	EHA105	pCAMBIA2301	*GUS*	OD600 = 0.8, 200 μM AS, 30 min infection duration co-culture 3d	/	Wei, 2018
*P. lactiflora* ‘Duchesse de Nemours’	*Arabidopsis thaliana* flowers	Stable Genetic Transformation	infection	GV3101	pTRV2	*GFP*	OD600 = 0.8, 800 μM Silwet L-77, 20 min infection durationt, 18 h infection duration	Plant attributes- senescence	Sun et al., 2025
*P. rockii* 'Jinghelan'	*Arabidopsis thaliana* seeds	Stable Genetic Transformation/Transient Genetic Transformation	infection/ VIGS	GV3101	pBI101/pTRV2	*GFP*	OD600 = 0.8, 800 μM Silwet L-77, 25 min infection durationt, 12 h infection duration	Plant attributes- promotes seed oil accumulation	Zhang et al., 2026
*P. ostii*	seed embryos	Transient Genetic Transformation	VIGS	GV3101	PoABI5	*GFP*	OD600 = 1.0, 200 μM AS, 6 negative pressure treatments, 2 h infection duration	/	Zhai et al., 2025
*P. lactiflora* ‘Fen Yu Nu’×’Xi Shi Fen’	callus	Transient Genetic Transformation	infection	EHA105	pBI121	*GUS*	OD600 = 0.6, 100 μM AS, 0.9 MPa, 20 min treatments, co-culture 3d	/	Duan et al., 2022
*P. lactiflora* ‘WHLY’	petals	Transient Genetic Transformation	infection/ VIGS	GV3101	pCAMBIA1300/ pTRV1	*GFP*	OD600 = 0.6, 100 μM AS, 0.9 MPa, 15 min treatments, 3 h infection duration	Plant attributes- geraniol biosynthesis	Zhao Q. et al., 2025
*P. ostii*	buds	Transient Genetic Transformation	VIGS	GV3101	PoFBA5	*GFP*	OD600 = 0.8, 100 μM AS, 0.1 MPa, 20 min treatments, 1 h infection duration	Stress resistance— drought	Luan et al., 2024
*P. lactiflora* ‘Hang Baishao’	one-year-old roots	Transient Genetic Transformation	VIGS	GV3101	PlHB31	*GFP*	OD600 = 1.5, 200 μM AS, negative pressure treatments, 3 h infection duration	Plant attributes- dormancy	Zhang et al., 2024
*P. rockii*	seedlings	Transient Genetic Transformation	VIGS	GV3101	pCAMBIA2300	*GFP*	OD600 = 1.0, 200 μM AS, 0.1 MPa, 20 min treatments, 48 h infection duration	Plant attributes- seed oil biosynthesis	Yang et al., 2023
*P. ostii* ‘Feng Dan’	seedlings	Transient Genetic Transformation	VIGS	GV3101	PoPDS	*GFP*	OD600 = 1.0, 200 μM AS, 0.1 MPa, 20 min treatments, 48 h infection duration	Plant attributes-	Xie et al., 2019
*P. suffruticosa* ‘Taiyoh’, ‘Hu Hong’	petals	Transient Genetic Transformation	gene gun	pSN1301	pCAMBIA1300	*GFP*	0.2 M sorbitol, 0.2 M mannitol, 6 h 9 μL of gene gun bullets, bombardment pressure 1100 psi, vacuuming to 26–30 inches mercury, bombardment the sample at a distance of 8 cm.	Plant attributes- Color	Wang Q. et al., 2023

Although a technical framework for the stable genetic transformation of *Paeonia* based on SE has been established, its development still faces many bottlenecks that restrict the large-scale application of this technology (Liu et al., 2013; Wang R. et al., 2025; Zhang et al., 2023b). On the one hand, the tissue culture system is insufficiently mature, and severe browning, difficulties in differentiation and rooting, and low regeneration efficiency in the regeneration system collectively hinder the advancement of transgenic *Paeonia* technology. Although some studies have attempted to address these problems through exogenous pretreatment, such as the addition of growth inhibitors and adjustment of medium and plant growth regulator types, Zhang et al. (2022) speculated that the undifferentiated state of the embryonic callus is related to high methylation, and the emergence of rooted plants may be associated with demethylation in *P. ostii*; genes such as *PoWOX*, *PoBBM*, and *PoGPT1* are hypothesized to promote SE and callus formation in *P. ostii* (Zhang et al., 2023c; Xia et al., 2022; Song et al., 2022). To date, preliminary propagation technologies based on tissue culture have only been established for *P. ostii* and *Paeonia × lemoinei* ‘High Noon’, and most cultivars still lack a stable tissue culture system (Zhang et al., 2023a; Zhang et al., 2022). On the other hand, the development of homologous transgenic systems is limited. In 2018, Wei (2018) obtained four complete *P. ostii* plants containing exogenous genes through *in vitro* regeneration using SE technology, for which the conversion rate was only 1.33%. However, this method has not been widely applied. With respect to *P. lactiflora*, although the *PlIpt* gene was introduced into the callus of the “Fen Yunu” cultivar via *Agrobacterium*-mediated transformation in 2007, the complete cultivation of transgenic plants was not achieved in the following years because of the lack of a mature tissue culture system (Wang et al., 2018; Wen et al., 2016a; Wen et al., 2021).

### 4.2 Transient genetic transformation

Owing to the greater challenges in the development of stable homologous transgenic systems for *Paeonia* plants, current research on gene function verification in *Paeonia* plants still relies mainly on heterologous transformation or homologous transient transformation systems (Song et al., 2022; Zhai et al., 2025; Zhang et al., 2024). From the perspective of technical essence and core advantages, transient genetic transformation of *Paeonia* is characterized by “transient expression of exogenous genes without integration into the host genome”. This characteristic not only prevents the potential interference of exogenous gene integration on the genetic stability of plants but also greatly shortens the research cycle (**Figures 2A, B**); the entire process from transformation to screening of the transformants only takes 1–2 weeks with a transient expression rate of up to 50–80%, creating a “rapid verifier” for research on the molecular mechanisms of *Paeonia* (Bian et al., 2020; Du et al., 2015).

In *Agrobacterium*-mediated transformation, the infiltration method (injection or soaking of somatic embryos) enables more extensive cell infection, and the optimization of key conditions further improves transformation efficiency. Guan et al. (2022) confirmed that when *Agrobacterium tumefaciens* was used for callus transformation, the addition of 0.01% Tween-20 under a negative pressure of 10 kPa to increase bacterial solution permeability, followed by cocultivation in the dark for 3 days, could effectively break through the resistance barrier of *Paeonia* tissues to *Agrobacterium*. This optimized protocol clarifies the core technical parameters for *Agrobacterium*-mediated *Paeonia* transient transformation and provides a reproducible and promotable operational standard for subsequent large-scale tissue-level transient transformation (Guan et al., 2022). The introduction of VIGS technology has further expanded the application boundary of transient genetic transformation in *Paeonia*, especially in the analysis of gene functions at the plant level. Xie et al. (2019) first applied Tobacco Rattle Virus (TRV)-mediated VIGS technology to *P. ostii* ‘Feng Dan’. By silencing the endogenous phytoene desaturase gene (*PoPDS*), the newly emerged leaves at the tops of the plants showed a typical photobleaching phenotype. Moreover, tracking the fluorescent signal of the TRV-GFP vector confirmed that the TRV virus could spread efficiently in different *Paeonia* tissues (such as leaves and roots) and achieve systemic gene silencing, laying a foundation for the application of VIGS technology in *Paeonia* (Xie et al., 2019). Later, Bian et al. (2020) used this technology to silence the *PlDELLA* gene in *P. lactiflora* and reported that the dormancy release process of plants accelerated and that the growth rate significantly increased. These findings clarify the negative regulatory role of the *PlDELLA* gene in regulating *Paeonia* dormancy and growth, fully verifying the effectiveness of VIGS technology in analyzing the functions of genes related to development in *Paeonia* (Bian et al., 2020). More progress was achieved by Zhang et al. (2024), who proposed an efficient homologous transient verification system. Through the use of one-year-old roots of *P. lactiflora* Pall. as materials, the authors precooled the specimens at 4 °C for 3–5 weeks as a key pretreatment and selected the dormancy-related gene *PlHB31* (which negatively regulates bud endodormancy release) as the target gene. This system not only resulted in “whole-plant” infiltration but also simplified the operation to the greatest extent possible. GFP fluorescence could be detected in newly formed roots and buds after direct infiltration; the bud germination of the transgenic plants significantly advanced, and the expression of *PlHB31* in the silenced plants significantly decreased. This system further improved the applicability and efficiency of VIGS technology in *Paeonia*. Taken together, these advances have progressively dismantled barriers to genetic manipulation in *Paeonia*, establishing a robust technical framework for functional genomics research and variety improvement in this economically important ornamental genus.

## 5 Conclusion and future perspectives

**Figure 3:**
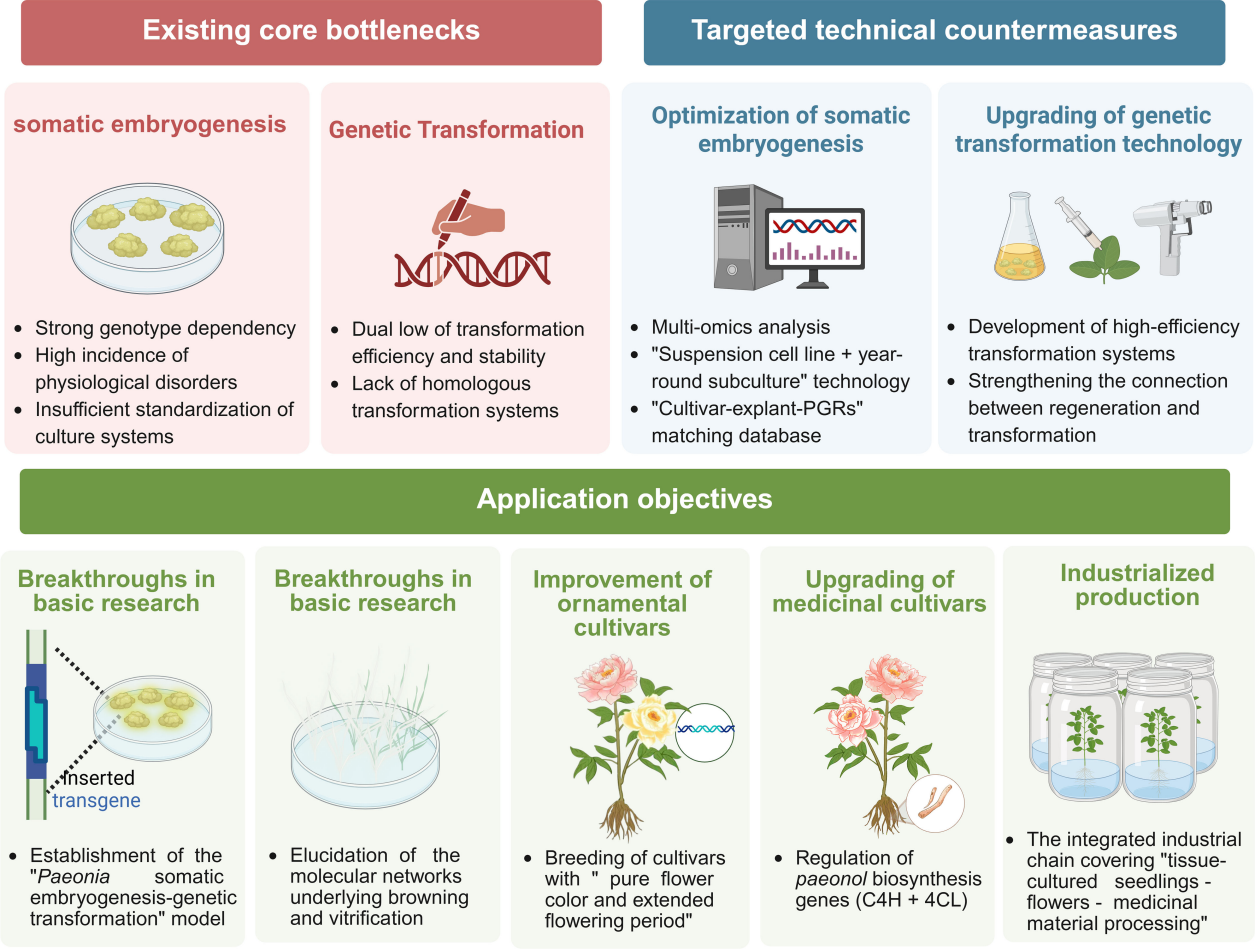
Bottleneck breakthroughs and future technologies for SE and genetic transformation in *Paeonia*.

This review systematically summarizes the key factors influencing SE efficiency in *Paeonia*, identifies critical bottlenecks hindering large-scale application, and evaluates the progress and challenges in genetic transformation via SE-mediated pathways (**Figure 3**). In the field of SE, genotype (e.g., low-heterozygosity cultivars such as ‘Feng Dan’ have an induction rate of up to 81.25%), explants (cotyledons and immature embryos are optimal), plant growth regulators (the optimal auxin/cytokinin ratio is 1:1.4–1:5), and culture conditions (23–25 °C for induction, 24–26 °C for maturation, low light or alternating red–blue light) are key factors that regulate efficiency. However, issues such as contamination, browning, hyperhydricity (with an incidence of up to 76.2% in some cultivars), and immature tissue culture systems still restrict large-scale application (Meng, 2011; Ren R. et al., 2019; Ci et al., 2022; Zhu et al., 2022; Song et al., 2023; Wen et al., 2025). Traditional SE culture in *Paeonia* relies heavily on manual operations, resulting in inconsistent standardization across laboratories. Bioreactor technology has been proposed as a potential solution (Jain et al., 2012), with the Growtek^®^ system demonstrating increased biomass production from nodal explants. The embryonic developmental stage should guide bioreactor selection: globular embryos, with fragile cell walls, benefit from gentle air-lift environments, whereas heart-shaped to torpedo-shaped embryos require optimized mass transfer for increased metabolic demand (Saha et al., 2020). However, bioreactors and automated platforms remain exploratory in *Paeonia*, with no reports of successful implementation to date. The development of integrated systems comprising automated disinfection, real-time culture monitoring (temperature, pH, and growth metrics), and machine vision-based embryo sorting may address scalability limitations, but these advances are contingent on first achieving reproducible SE in standard culture vessels.

In terms of genetic transformation, somatic embryos serve as ideal recipients because of their high degree of synchronization, stable regeneration, feasibility for large-scale culture, and low chimera rate. Stable genetic transformation relies mainly on the *Agrobacterium*-mediated method for exogenous gene integration, whereas the particle bombardment method is mostly used for preliminary preverification. Although the core value of “heritable traits and long-term genetic improvement” depends on the SE system, practical applications remain undefined at present. This is constrained by bottlenecks such as the immature tissue culture system of the *Paeonia* genus and strong genotype dependence (Wang et al., 2018; Wen et al., 2016a; Wen et al., 2021). In contrast, transient homologous transformation has become the mainstream method for gene function verification in this genus because of its short cycle and high efficiency (Yang et al., 2023; Zhang et al., 2026). While transient systems have enabled functional genomics studies—including analyses of senescence regulation (e.g., *PlPLATZ5*; Sun et al., 2025), dormancy release (e.g., PsmiR172b-*PsTOE3*; Zhang et al., 2023c);, metabolic optimization (e.g., *PoUGT84A1*; Kong et al., 2025), and stress responses (e.g., *PobZIP4*; Chen et al., 2024)—these achievements remain confined to model cultivars and cannot be generalized to commercial varieties.

Current research on SE and genetic transformation in the *Paeonia* genus still faces three key bottlenecks: (i) genotype-dependent recalcitrance, wherein protocols successful for ‘Feng Dan’ fail for most commercial cultivars; (ii) incomplete mechanistic understanding of critical barriers, including browning, hyperhydricity, and embryogenic transition failure; and (iii) the absence of stable transformation systems for all except *P. ostii*. Addressing these bottlenecks requires a multipronged strategy: (a) a mechanistic understanding of recalcitrance, wherein critical knowledge gaps remain regarding the molecular basis of genotype-dependent SE responsiveness; future studies should investigate the role of epigenetic modifications (e.g., DNA methylation) in regulating embryogenic competence, the interaction between hormone signaling pathways and key embryogenic genes (e.g., WUS, LEC, and BBM), and the molecular mechanisms underlying browning and susceptibility to hyperhydricity; (b) stable transformation system development, wherein the establishment of a genotype-independent transformation system is contingent on first achieving reproducible SE across cultivars, and until then, transient transformation remains the primary tool for functional genomics in responsive genotypes; (c) integration of emerging technologies, wherein gene editing (CRISPR/Cas9), multiomics approaches, and AI-assisted culture optimization offer promising avenues, but their successful application in *Paeonia* requires foundational improvements in SE efficiency first; and (d) scalability and industrial translation, wherein AI-assisted culture optimization (Cai et al., 2022) and automated platforms remain conceptual, and their development is premature without reproducible baseline protocols, while GMO safety evaluation systems and acclimatization protocols (targeting >90% survival) require established transformation systems as prerequisites. Thus, short-term priorities should focus on mechanistic research and protocol optimization rather than industrial scaling. These challenges are shared across many woody ornamental genera, but the exceptional recalcitrance of *Paeonia*—compared with that of model species such as *Rosa* or *Malus*—suggests that dedicated research investment is needed to achieve parity with other high-value crops.
